# Arachidonic Acid Metabolites of CYP450 Enzymes and HIF-1α Modulate Endothelium-Dependent Vasorelaxation in Sprague-Dawley Rats under Acute and Intermittent Hyperbaric Oxygenation

**DOI:** 10.3390/ijms21176353

**Published:** 2020-09-01

**Authors:** Zrinka Mihaljević, Anita Matić, Ana Stupin, Ruža Frkanec, Branka Tavčar, Vanja Kelava, Ivana Tartaro Bujak, Nikolina Kolobarić, Aleksandar Kibel, Ines Drenjančević

**Affiliations:** 1 Institute and Department of Physiology and Immunology, Faculty of Medicine Osijek, Josip Juraj Strossmayer University of Osijek, J. Huttlera 4, 31000 Osijek, Croatia or zmihaljevic@mefos.hr (Z.M.); acosic@mefos.hr (A.M.); ana.stupin@mefos.hr (A.S.); nbdujmusic@mefos.hr (N.K.); aleksandar_mf@yahoo.com (A.K.); 2 Scientific Center of Excellence for Personalized Health Care, Josip Juraj Strossmayer University of Osijek, Trg Svetog Trojstva 3, 31000 Osijek, Croatia; 3 Department of Pathophysiology, Physiology and Immunology, Faculty of Dental Medicine and Health Studies, Josip Juraj Strossmayer University of Osijek, Crkvena ul. 21, 31000 Osijek, Croatia; 4 Centre for Research and Knowledge Transfer in Biotechnology, University of Zagreb, Trg Republike Hrvatske 14, 10000 Zagreb, Croatia; ruza.frkanec@unizg.hr; 5 Department of Drug Metabolism and Pharmacokinetics, Fidelta Ltd., Prilaz baruna Filipovića 29, 10000 Zagreb, Croatia; Branka.Tavcar@fidelta.eu (B.T.); Vanja.Kelava@fidelta.eu (V.K.); 6 Radiation Chemistry and Dosimetry Laboratory, Division of Materials Chemistry, Ruđer Bošković Institute, Bijenička cesta 54, 10000 Zagreb, Croatia; itartaro@irb.hr; 7 Department of Heart and Vascular Diseases, Osijek University Hospital, J. Huttlera 4, 31000 Osijek, Croatia

**Keywords:** acetylcholine, aortic rings, epoxyeicosatrienoic acids, hyperbaric oxygenation, hypoxia, HIF-1α

## Abstract

Acetylcholine-induced vasorelaxation (AChIR) and responses to reduced pO_2_ (hypoxia-induced relaxation (HIR), 0% O_2_) were assessed in vitro in aortic rings of healthy male Sprague-Dawley rats (N = 252) under hyperbaric (HBO_2_) protocols. The studied groups consisted of the CTRL group (untreated); the A-HBO_2_ group (single HBO_2_; 120 min of 100% O_2_ at 2.0 bars); the 24H-HBO_2_ group (examined 24 h after single exposure) and the 4D-HBO_2_ group (four consecutive days of single HBO_2_). AChIR, sensitivity to ACh and iNOS expression were decreased in the A-HBO_2_ group. HIR was prostanoid- and epoxyeicosatrienoic acid (EET)-mediated. HIF-1α expression was increased in the 24H-HBO_2_ and 4D-HBO_2_ groups. LW6 (HIF-1α inhibitor) decreased HIR in the 24H-HBO_2_ group. HBO_2_ affected the expression of COX-1 and COX-2. CYP2c11 expression was elevated in the 24H-HBO_2_ and 4D-HBO_2_ groups. Concentrations of arachidonic acid (AA) metabolites 14(15)-DiHET, 11(12)-DiHET and 8(9)-DiHET were increased in A-HBO_2_ and 24H-HBO_2._ An increased concentration of 8(9)-EET was observed in the A-HBO_2_ and 24h-HBO_2_ groups vs. the CTRL and 4D-HBO_2_ groups, and an increased concentration of 5(6)-DiHET was observed in the 24H-HBO_2_ group vs. the 4D-HBO_2_ group. The 20-HETE concentration was increased in the A-HBO_2_ group. All were determined by LC-MS/MS of the aorta. The results show that AChIR in all groups is mostly NO-dependent. HIR is undoubtedly mediated by the CYP450 enzymes’ metabolites of AA, whereas HIF-1α contributes to restored HIR. Vasoconstrictor metabolites of CYP450 enzymes contribute to attenuated AChIR and HIR in A-HBO_2_.

## 1. Introduction

Although the beneficial effects of hyperbaric oxygenation (HBO_2_) on tissue perfusion are well documented, there is a paucity of knowledge about the mechanisms by which HBO_2_ improves tissue oxygenation. It is known that various arachidonic acid metabolites (prostaglandins, epoxyeicosatrienoic acids (EETs), hydroxy-eicosatrienoic acids (HETEs)) and NO are of the utmost importance in mediating vascular reactivity to vasodilators and vasoconstrictors [1,2,3,4,5], including hypoxia and hyperoxia stimuli [6]. In conditions of reduced blood flow, the use of HBO_2_ can significantly increase tissue oxygenation. Intermittent exposure to HBO_2_ has been shown to cause activation of the CYP450 epoxygenase pathway in the macrovasculature of diabetic animals and to restore vasorelaxation of aortic rings in response to acetylcholine [7]. Furthermore, Kibel et al. (2012; 2015) showed an improved relaxation response to ANGII and ANG- (1–7) in healthy animals treated by intermittent HBO_2_ due to activation of mechanisms related to CYP450 enzyme activation and EET synthesis [8,9]. However, the way in which acute or intermittent HBO_2_ affects the mechanisms of vasodilation to physiological stimuli such as reduced pO_2_ or acetylcholine (ACh) in healthy blood vessels is still not known. Since HBO_2_ has been increasingly used both for health (e.g., sport) treatments and cosmetic purposes, understanding the mechanisms of action and consequences of the utilization of HBO_2_ in healthy individuals is of primary importance.

Endothelial cells metabolize arachidonic acid (AA) via CYP450 enzymes (ω-hydroxylase and epoxygenase), via cyclooxygenases and 5-lipooxygenase, and by non-enzymatic degradation of arachidonic acid in the presence of free radicals [10]. Epoxygenases are members of cytochrome P450 family of enzymes, which in the endothelial cell produce four epoxyeicosatrienoic acid (EET) isomers, of which 14,15-EET and 11,12-EET are the major active vasodilator metabolites [10,11,12,13,14,15]. ω-hydroxylase, in smooth muscle cells, promotes the production of 20-hydroxy-eicosatrienoic acid (20-HETE), which is a vasoconstrictor [15,16]. Two isoforms of cyclooxygenase (COX-1 and COX-2) are involved in the synthesis of prostanoids from AA. The resulting prostanoids act antagonistically, causing vasodilation (prostaglandin D_2_, prostaglandin E_2_ and prostacyclin I_2_) and vasoconstriction (prostaglandin F_2α_ and thromboxane A_2_). Hypoxia activates COX-1 to produce prostacyclin (PGI_2_), which promotes the opening of several types of potassium channels via cAMP, resulting in hyperpolarization of the smooth muscle membrane and consequent vasodilation [10]. Increased oxygen delivery significantly activates CYP450 enzymes and increases the production of 20-HETE, and also EET [17].

Recently, we showed that increased superoxide production and an overall increase in oxidative stress impairs vasorelaxation in rats acutely exposed to HBO_2_, whereas intermittent exposure to HBO_2_ increases the expression and activity of antioxidative enzymes, suggesting that intermittent HBO_2_ induces antioxidative defense mechanisms underlying restored vasorelaxation [18]. Altogether, there is no clear answer as to which mediators are involved in the vasoconstriction or vasorelaxation observed in various HBO_2_ protocols, because up to the present study, no measurements of vascular eicosanoids production have yet been performed.

The role of transcription factor hypoxia inducible factor 1 alpha (HIF-1α) in the effects of HBO_2_ was described in studies on wound healing (diabetic foot, postiradiation injury). HIF-1α is involved in neovascularization, cell proliferation and increased mobilization of progenitor endothelial cells from bone marrow [19,20]. HBO_2_ activates HIF-1α at several levels by increasing both HIF-1α stability and activity [21]. Hypoxia and reactive oxygen species (ROS)/ reactive nitrogen species (RNS) may stimulate HIF-1α stabilization, leading to the activation of hypoxia-induced cellular signaling pathways [22]. There is a limited knowledge on the role of HIF-1α on vascular reactivity in conditions of elevated oxidative stress and exposure to HBO_2_.

Taken together, the hypothesis of the present study was that HBO_2_ changes the mechanisms of vascular relaxation and affects HIF-1α expression. Furthermore, HIF-1α is involved in vascular relaxation mechanisms, depending on the duration and frequency of HBO_2_ exposure. Therefore, the purpose of this study was to investigate whether the mechanisms of ACh- and hypoxia-induced vascular relaxation in healthy rats and the expression of HIF-1α in aortic tissue were influenced by acute and intermittent HBO_2_. A further goal was to determine the involvement of metabolites of AA, produced in CYP450 enzymatic pathways, in vasorelaxation in different HBO_2_ protocols.

## 2. Results

### 2.1. Body Mass and Blood Pressure of Studied Groups

Body mass (g) of rats was not significantly different among examined groups. There was a significant decrease in mean arterial pressure in the A-HBO_2_ group, compared to other groups (Table 1).

### 2.2. Acetylcholine-Induced Vasorelaxation (AChIR) of Isolated Rat Aortic Rings

**Figure 1:**
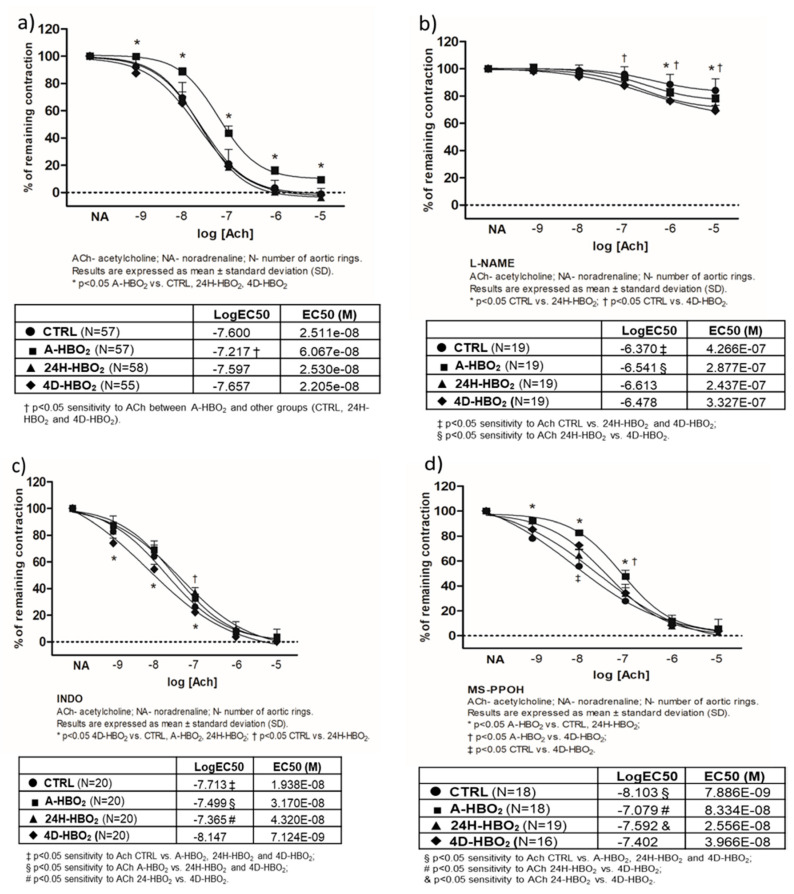
Ach-induced relaxation (AChIR) of isolated rat aorta rings in the CTRL, A-HBO_2_, 24H-HBO_2_ and 4D-HBO_2_ groups. Baseline relaxation to acetylcholine (**a**) and relaxation to acetylcholine in the presence of eNOS inhibitor L-NAME (**b**), COX inhibitor INDO (**c**) and a selective CYP450 epoxidase inhibitor, MS-PPOH (**d**) in CTRL, A-HBO_2_, 24H-HBO_2_ and 4D-HBO_2_ groups of rats. Results are presented as mean ± SD; N—number of aortic rings; *p* < 0.05.

Figure 1a presents the results of isolated aortic ring baseline vasorelaxation in response to ACh (AChIR) in all experimental groups of rats. AChIR was significantly reduced in the A-HBO_2_ group (the group receiving a single HBO_2_ exposure) compared to all other groups of animals. A-HBO_2_ rats also exhibited lower sensitivity to ACh compared to other groups of rats (presented by logEC50 in tables). HBO_2_ significantly affected AChIR by reducing the inhibition of vasorelaxation caused by L-NAME compared to the control (CTRL) group, although sensitivity to ACh was not significantly different (Figure 1b).

The role of COX-1 and COX-2 metabolites was assessed with indomethacin. In the 4D-HBO_2_ group (receiving four consecutive days of single HBO_2_ exposure), AChIR was significantly improved after indomethacin compared to the other groups. In the 24H-HBO_2_ group (examined 24 h after single exposure) indomethacin partially inhibited AChIR at a dose of 10^−7^ mol L^−1^ compared to the CTRL group (Figure 1c). Furthermore, MS-PPOH, an inhibitor of epoxygenase reactions significantly inhibited AChIR in the A-HBO_2_ group compared to the CTRL and 24H-HBO_2_ groups at ACh concentrations of 10^−9^–10^−7^ mol L^−1^, and compared to the 4D-HBO_2_ group at 10^−7^ mol L^−1^. MS-PPOH partially inhibited AChIR at 10^−8^ mol L^−1^ in the 4D-HBO_2_ group versus the CTRL group (Figure 1d).

**Figure 2:**
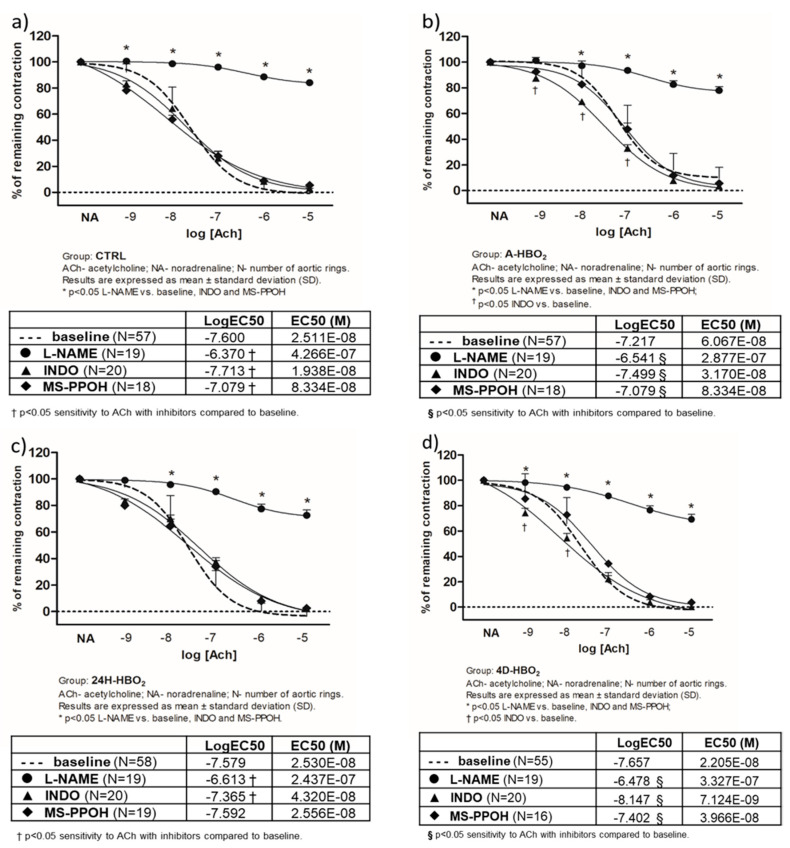
Mechanisms of AChIR response of isolated rat aorta rings in CTRL (**a**), A-HBO_2_ (**b**), 24H-HBO_2_ (**c**) and 4D-HBO_2_ (**d**) groups of rats with and without inhibitors (L-NAME, INDO, MS-PPOH). Results are presented as mean ± SD; N—number of aortic rings; *p* < 0.05.

Figure 2 presents the mechanisms of AChIR in each experimental group of rats. Administration of L-NAME eliminated AChIR in the CTRL, A-HBO_2_ and 24H-HBO_2_ groups. In the 4D-HBO_2_ group, the AChIR of the aortic rings was partially inhibited by the use of L-NAME and partially inhibited by MS-PPOH at ACh doses of 10^−6^ mol L^−1^ and 10^−7^ mol L^−1^ (Figure 2d).

The sensitivity to ACh (presented by logEC50 in the tables) decreased with L-NAME in the CTRL group. In the A-HBO_2_ group (Figure 2b), indomethacin increased the sensitivity of aortic rings to ACh, whereas L-NAME and MS-PPOH decreased the sensitivity to ACh compared to the baseline response. In the 24H-HBO_2_ group (Figure 2c), ACh sensitivity was mostly reduced by L-NAME compared to the baseline response. MS-PPOH and indomethacin also reduced the sensitivity to ACh compared to the CTRL group. In the 4D-HBO_2_ group, ACh sensitivity was also decreased in the presence of L-NAME, and increased in the presence of indomethacin and MS-PPOH (Figure 2d).

### 2.3. Hypoxia-Induced Vasorelaxation of Isolated Rat Aortic Rings

**Figure 3:**
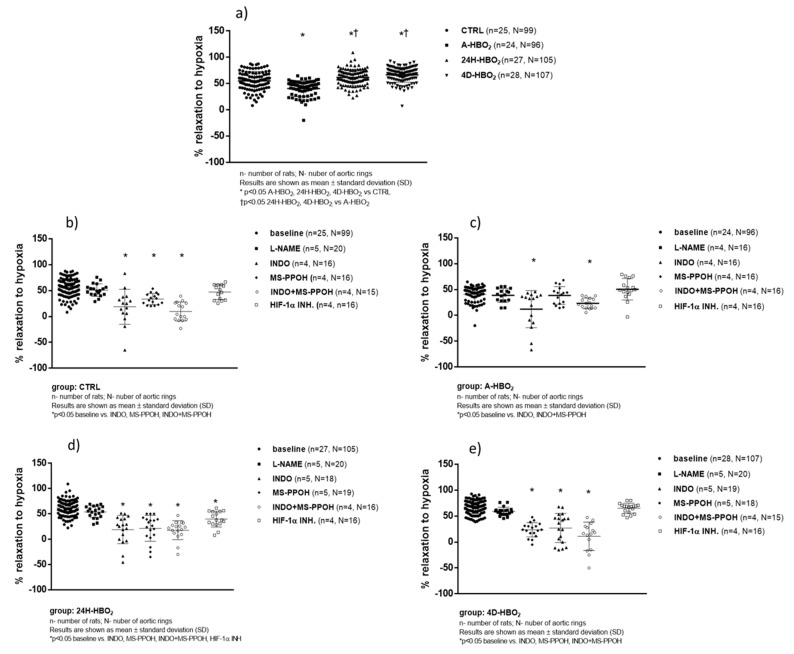
Hypoxia-induced relaxation (HIR) response of isolated rat aorta rings in CTRL (**b**), A-HBO_2_ (**c**), 24H-HBO_2_ (**d**) and 4D-HBO_2_ (**e**) groups without (**a**) and with inhibitors L-NAME, INDO, MS-PPOH, INDO + MS − PPOH and HIF-1α inhibitor − LW6. Results are presented as mean ± SD; n—number of rats, N—number of aortic rings; * *p* < 0.05 compared to baseline.

The A-HBO_2_ group exhibited significantly decreased baseline vasorelaxation in response to hypoxia (hypoxia-induced relaxation (HIR)) compared to all other groups of rats. The 24H-HBO_2_ and 4D-HBO_2_ groups exhibited significantly enhanced HIR compared to the CTRL and A-HBO_2_ groups of rats (Figure 3a). HIR was significantly lower in the presence of INDO, MS-PPOH and INDO + MS-PPOH compared to the baseline in the CTRL and 4D-HBO_2_ groups (Figure 3b,e). INDO eliminated HIR in A-HBO_2_, whereas MS-PPOH and HIF-1α inhibitor did not have a significant effect in that group (Figure 3c). The combination of INDO and MS-PPOH significantly reduced the relaxation in the A-HBO_2_ group compared to the baseline, similar to that of INDO per se. In the 24h-HBO_2_ group (Figure 3d) significant inhibition of HIR was achieved in the presence of INDO and MS-PPOH individually, in the presence of both INDO + MS − PPOH and in the presence of LW6 (HIF-1α inhibitor) compared to the baseline response. The use of L-NAME had no effect on the relaxation response to hypoxia in any of tested groups (Figure 3b–e).

### 2.4. Relative mRNA and Protein Expression of Enzymes in Rat Aorta

**Figure 4:**
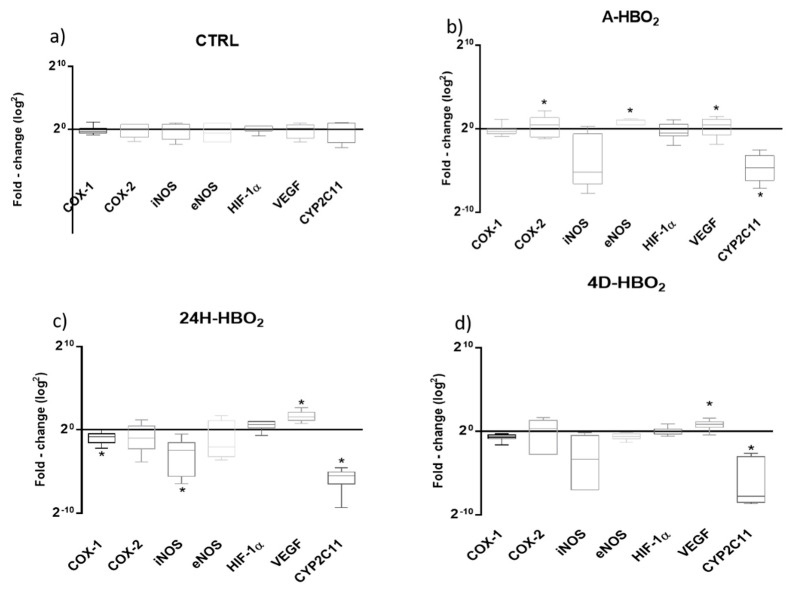
Expressions of COX-1, COX-2, iNOS, eNOS, HIF-1α, VEGF and CYP2C11 in rat aorta in CTRL (**a**), A-HBO_2_ (**b**), 24H-HBO_2_ (**c**) and 4D-HBO_2_ group (**d**). Results were normalized to *HPRT* housekeeping gene and control and presented using whisker bars as fold change (2^−ΔΔCT^). * *p* < 0.05 was considered significant.

**Figure 5:**
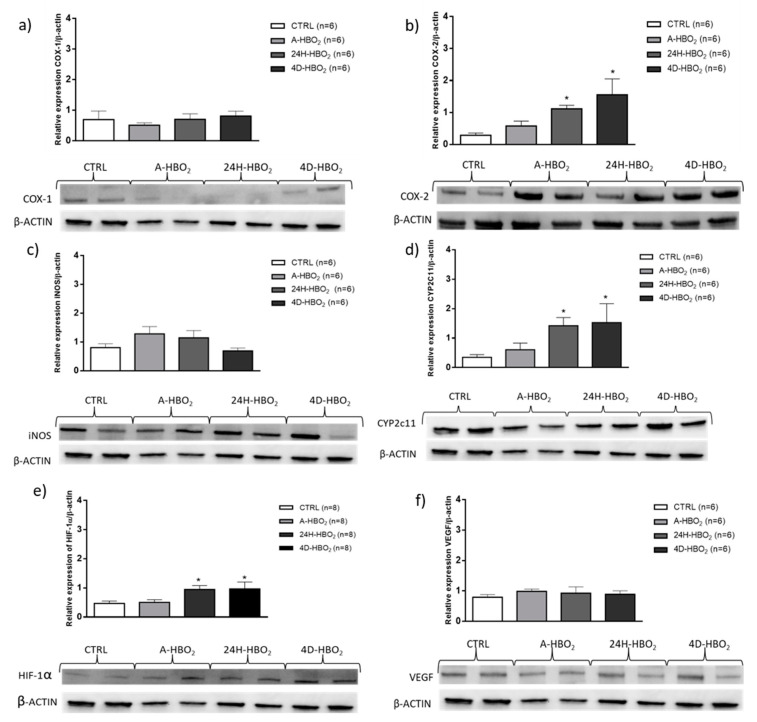
Relative protein expression and representative blots of (**a**) COX-1—cyclooxygenase 1; (**b**) COX-2—cyclooxygenase 2; (**c**) iNOS—inducible nitric oxide synthase; (**d**) CYP2C11; (**e**) HIF-1α and (**f**) VEGF in aortic tissue, determined by Western blot method. Results are shown as mean ± standard deviation (SD); * *p* < 0.05 compared to CTRL.

Relative mRNA expression of COX-1 was significantly reduced only in the 24H-HBO_2_ group, compared to other groups (Table 2, Figure 4). mRNA expression of COX-2 was significantly reduced in the 4D-HBO_2_ group, whereas protein expression of COX-2 was significantly increased compared to controls (Table 2, Figure 5b). COX-2 mRNA was also significantly reduced in the 24H-HBO_2_ and 4D-HBO_2_ groups, compared to the A-HBO_2_ group. The relative mRNA expression of iNOS was significantly reduced in the A-HBO_2_ and 24H-HBO_2_ groups, compared to the control group, whereas eNOS expression was not significantly different among the groups (Table 2, Figure 4).

Relative expression of CYP2c11 mRNA was significantly elevated in the 4D-HBO_2_ group, compared to other groups (Table 2), whereas CYP2c11 protein expression was significantly increased in 24H-HBO_2_ and 4D-HBO_2_ compared to the CTRL group (Figure 5d). Other investigated proteins were similarly expressed among groups (Figure 5).

### 2.5. HIF-1α mRNA and Protein Expression

Relative gene expression of HIF-1α transcription factor and its target gene VEGF was significantly increased in the 24H-HBO_2_ and 4D-HBO_2_ groups, compared to the CTRL and A-HBO_2_ groups (Table 2), fold change (2^−ΔΔCT^) didn’t showed significant changes (Figure 4). Relative protein expression of HIF-1α was significantly increased in 24H-HBO_2_ and 4D-HBO_2_, compared to the CTRL group (Figure 5e).

### 2.6. Liquid Chromatography-Tandem Mass Spectrometry Determination of CYP450 Enzymatic Pathway Metabolites of Arachidonic Acid

LC/MS-MS of metabolites of arachidonic acid, produced in CYP450 enzymatic pathways (Table 3), in aorta samples showed significantly increased concentrations of vasodilatory EETs or their corresponding biodegradation products (DiHETs) as follows: increased concentrations of 14(15)-DiHET, 11(12)-DiHET and 8(9)-DiHET in A-HBO_2_ and 24 h-HBO_2_ groups versus CTRL; increased concentration of 8(9)-EET in A-HBO_2_ and 24 h-HBO_2_ groups vs. CTRL and 4D-HBO_2_ groups; and increased concentration of 5(6)-DiHET in 24h-HBO_2_ group vs. 4D-HBO_2_ group.

Determination of vasoconstrictory 20-HETE showed its increased concentration in the A-HBO_2_ group compared to CTRL and 4D-HBO_2_ groups. There was a significantly decreased ratio of 14(15)-EET to 20-HETE, 11(12)-DiHET to 20-HETE and 8(9)-EET to 20–HETE in the A-HBO_2_ group compared to other groups (CTRL, 24h-HBO_2_ and 4D-HBO_2_), and an increased ratio of 11(12)-DiHET to 20-HETE in the 4D-HBO_2_ group versus the CTRL and A-HBO_2_ groups (Table 4).

## 3. Discussion

The most important findings in the present study are: (a) a decrease in the contribution of NO to ACh-induced relaxation in HBO_2_-exposed animals; (b) improved hypoxia-stimulated relaxation after HBO_2_ (24 h after single exposure and intermittent HBO_2_); (c) for the first time, a demonstration that HBO_2_ may increase production of 20-HETE and EETs in aortic tissue and increase the ratio of vasodilatory/vasoconstrictor metabolites (after intermittent HBO_2_); (d) increased synthesis of HIF-1α and its involvement in hypoxia-stimulated relaxation of rat aortic rings, and (e) confirmation of EETs as endothelium-derived hyperpolarizing factors (EDHFs) and epoxygenases as oxygen sensors in HBO_2_.

Recently, our research group [7,8,9] showed the engagement of alternative pathways of endothelium-dependent relaxation in response to acetylcholine and ANG (1–7) in diabetic animals exposed to four days of HBO_2_. Interestingly, some previous studies have shown that if HBO_2_ is applied more than once, adaptive mechanisms are activated to protect against further oxidative damage, e.g., increased antioxidative protection and general prevention against the genotoxic action of H_2_O_2_, mediated by increased protection by intracellular antioxidants of leukocytes and antioxidants, which scavenge ROS that are distant from nuclear DNA [23,24]. Consequently, HBO_2_ preconditioning can be used for the prevention of subsequent oxidative injuries [25,26]. These HBO_2_-triggered adaptive and preconditional responses could also be responsible for changes in the underlying vascular reactivity mechanisms [7,8,9,27]. Since the effectiveness of HBO_2_ treatments generally depends on exposures being repeated within a few days [28], it is especially important to define HBO_2_’s molecular interactions when repeatedly applied.

### 3.1. Body Mass and Blood Pressure of Studied Groups

The observed decrease in mean arterial pressure is consistent with our previous studies, which discuss this issue [18,29]. Oxidative stress induced impaired baseline AChIR and HIR in the A-HBO_2_ group, which was also previously described and discussed in our previous study [18].

### 3.2. The Mechanisms of Acetylcholine-Induced Vasorelaxation 

The results show a decrease in the contribution of NO to ACh-induced relaxation in HBO_2_-exposed animals, despite the increased sensitivity of vascular smooth muscles to NO, which appears to be proportional to the duration of HBO_2_ treatment (Figure 2b), as well as the equal contribution of COX-produced prostacyclin and CYP450 metabolites of AA in hypoxia-stimulated vascular relaxation (Figure 3). As mentioned above, our previous findings, presented by Unfirer et al. and Kibel et al. [7,8,9], also showed the presence of alternative pathways of endothelial relaxation to acetylcholine and ANG (1–7) in diabetic animals exposed to four days of HBO_2_, which represent the most likely avenues of increase in production or sensitivity to EETs. Most importantly, the discovery of the participation of CYP450 metabolites in vasorelaxation in HBO_2_ is a novelty of the present study. These metabolites have been measured here for the first time ever in aortic tissue after HBO_2_ treatment.

### 3.3. The Mechanisms of Hypoxia-Induced Vasorelaxation 

A major part of the vasodilation response to hypoxia is mediated by the activation of COX and the consequent production of prostacyclin (PGI_2_), which then activates K_ATP_ channels [30]. Frisbee et al. [31] have shown that hypoxic vasodilation of skeletal muscular resistance arteries activates the cytochrome P450 enzyme pathway and that the hypoxic vasodilation response is not exclusively dependent on the COX pathway and prostacyclin production, but rather that EETs play a role in vasodilation in response to hypoxia. Some authors have shown that blood vessels with EDHF responses (coronary, cerebral and pulmonary) have a preserved EDHF response under severe hypoxia conditions, for which CYP450 epoxygenase is also responsible [32].

In the present study, the control group had an expected vasodilating response to hypoxia, whereas HIR was impaired after a single exposure to HBO_2_. Furthermore, 24 h after HBO_2_ and after four days of repeated HBO_2_ treatments (i.e., intermittent exposures), an increased vasodilating response to hypoxia appeared, suggesting that HBO_2_ led to a change in the activation and/or expression of COX and/or CYP450 enzymes (Figure 3a). The vasodilation response of the control aorta was fully mediated by activation of COX and PGI_2_ formation, because vasodilation was almost completely blocked after incubation with indomethacin. MS-PPOH reduced vasodilation at half the value of the baseline, indicating that CYP450 epoxygenase and the formation of EETs play a smaller role in vasodilation in hypoxia (Figure 3b). Twenty-four hours after single exposure to HBO_2_ and after four consecutive days, incomplete inhibition of vasodilation occurs after the administration of indomethacin. There is an even greater inhibitory effect on vasodilation after the administration of MS-PPOH, indicating that HBO_2_ induced higher EET production. Overall, these functional results suggest an important switch from the production of COX metabolites to CYP450 vasodilator metabolites after intermittent HBO_2_ exposure.

The particular significance of present work is that we have measured eicosanoid production in aortic tissue for the first time ever. These measurements (Table 3 and Table 4) support the results of functional studies with inhibitors. Acute HBO_2_ exposure increased 20-HETE formation, which led to attenuated vasorelaxation in response to ACh and hypoxia. This explains the restored vasorelaxation in 4D-HBO_2_ group and the altered mechanisms of vasorelaxation (with increased ratios of vasodilator to vasoconstrictor metabolites of CYP450 enzymes in this group), indicating that the vasodilation mechanisms are diverted to EETs. This is further supported by increased CYP2c11 gene and protein expression and an increased ratio of vasodilatory AA metabolites. Our results show increased concentrations of vasoconstrictor 20-HETE and a decreased ratio between various vasodilatory metabolites and 20-HETE in the A-HBO_2_ group, but also show increased concentrations of vasodilatory metabolites in the A-HBO_2_ and 24H-HBO_2_ groups. Altogether, our results confirm epoxygenase and omega-hydroxylase as oxygen sensors, and reveal for the first time that EETs are the mediators responsible for recovery of vasorelaxation after intermittent HBO_2_ treatment. Furthermore, the present study, with its direct measurements of CYP450 metabolites, confirms our hypothesis that periods between two hyperbaric exposures can be considered pseudohypoxia, which is a stimulus to enzyme upregulation and the alternation of vasoactive pathways [6,29]. The question of whether this is influenced by HIF-1α is discussed below.

### 3.4. The Role of HIF-1α in Vasorelaxation in Response to ACh and Hypoxia

Activation of HIF-1 involves redox-dependent stabilization of HIF-1α proteins [33]. In hypoxia, HIF-1α is translocated in the core and heterodimerized with a β subunit, forming the HIF-1 complex [34]. Two distinct domains within the HIF-1α subunit are responsive to cellular oxygen levels [35]. Expression and activation of the HIF-α subunits is firmly regulated, and their degradation by ubiquitin proteasome usually occurs in hyperoxic conditions [36,37]. However, independently of hypoxic or normoxic conditions, free radicals are needed to express HIF-α [36,37]. In acute HBO_2_ exposure, the oxidative stress is increased [18]. Previously, it has been shown that HIF-1 and -2 levels are increased in HBO_2_ treatment due to increased ROS production [37,38]. Using a DNA microarray, it has been reported that more than 2% of all human genes in arterial endothelial cells are regulated by HIF-1α, directly or indirectly [39], including genes for vascular reactivity and structural responses. For example, HIF-1α affects the expression of eNOS, iNOS, HO-1, COX-2, and the production of NO and prostaglandin under hypoxic conditions, such as hemorrhagic shock [40]. Importantly, changes in partial oxygen pressure modulate the synthesis of arachidonic acid metabolites [17,41]. The synthesis of epoxyeicosatrienoic acids (EETs), and also of 20-hydroxysacetoxyetraenic acids, is pO_2_-dependent and reaches its maximal plateau level at pO_2_ from 80–150 mmHg. At lower values of pO_2_ the formation of 20-HETE and EETs is reduced and is linearly dependent on pO_2_ between 20 and 140 mm Hg, but the slope is less steep for EETs [17]. Whether HIF-1α has a role in changes of AA metabolite production in hyperbaric oxygenation [41] is still unclear, since there are contradictory results regarding HIF-1α expression in conditions of increased pO_2_, with various protocols of HBO_2_ exposure [42,43,44,45]. However, in our functional experiments on aortic rings, we demonstrated the role of HIF-1α in the restored HIR. The blockade of HIF-1α expression attenuated HIR.

As already explained, the results of this study support our earlier hypothesis on the occurrence of pseudohypoxic conditions in pauses between two exposures. Although *COX-1* and *-2* gene expression decreased in the 24H-HBO_2_ and 4D-HBO_2_ groups, the protein expression of COX-2 (Figure 5b) was increased, in accordance with the results of Kaidi et al. [46], who demonstrated that the upregulation of COX-2 is transcriptional and is associated with induction by HIF-1α. Results also indicate the association of HBO_2_, hypoxia, HIF-1α and increased EET production. Such results are consistent with the findings of Chen and Goldstein [47], suggesting a positive feedback mechanism that can explain induction and activation of CYP2C during hypoxia, although it remains unclear how EETs increase the expression of HIF-1α proteins and how phosphorylated AMPK activates transcription of the *CYP2C* gene. Suzuki and colleagues [39] have shown that the expression of mRNA HIF-1α does not increase with EETs and suggest that EETs stabilize HIF-1α by activating the PI3K/Akt path to induce the expression of VEGF. Taken together, the results of the present study suggest that improved relaxation in pseudohypoxic conditions after HBO_2_ (after one exposure or four days of exposures) are in line with these studies. Functional results in the present study show the involvement of EETs in relaxation itself. It is plausible that HBO_2_, by increasing tissue pO_2_, induces oxidative stress. This oxidative stress consequently activates HIF-1α, as well as CYP2C11, which is followed by increased EET synthesis, which stabilizes HIF-1α. This further activates COX-2, to produce more prostacyclin and thus improve relaxation. Additionally, increased production of EETs or corresponding DiHETs serves as a substrate to increase COX activity. Once formed, epoxyeicosatrienoic acids (EETs) and 20-hydroxyeicosatetraenoic acid (HETE) are metabolized by β-oxidation to 18- and 16-carbon derivatives, which are less biologically active [48,49,50]. 5,6-EET, 8,9-EET and 20-HETE can also be metabolized by cyclooxygenase (COX) to vasoconstrictor endoperoxides or to vasodilator prostaglandin or prostacyclin-like derivatives [51]. Altogether, we have confirmed our hypothesis regarding the involvement of HIF-1α in HBO_2_ effects. The importance of the present study is that results are highly conclusive in terms of the contribution of the metabolites of arachidonic acid (produced via CYP450 pathways) in the altered mechanisms of vasorelaxation. The present study demonstrates the importance of EETs and 20-HETE in vasorelaxation in healthy experimental animals, although we have also previously demonstrated their role in conditions such as stroke [52,53]. The present study lays the groundwork for the transition to human studies considering the mechanisms of the beneficial effects of HBO_2_ in stroke.

## 4. Materials and Methods

### 4.1. Hyperbaric Oxygenation Exposure Protocols

The animals were bred and housed at the animal care facility of the Faculty of Medicine, Osijek. All experimental procedures conformed to the European Convention for the Protection of Vertebrate Animals Used for Experimental and other Scientific Purposes (Council of Europe No 123, Strasbourg 1985) and the European Guidelines for the Care and Use of Laboratory Animals (directive 86/609) and were approved by the local and national Ethical Committee (#2158/61-02-139/2-06).

A total of 252 male Sprague-Dawley (SD) rats (aged 9–12 weeks) were used in this study. Rats were housed at a temperature of 21 °C–23 °C), in a humidity- and light-controlled room with free access to tap water and were fed ad libitum with a commercially prepared pellet diet (Mucedola, Italy). Hyperbaric groups underwent 120 min daily sessions of 100% O_2_ at 2.0 bars absolute pressure at a flow rate of 2–3 L/min, with an additional 15 min for gradual compression and decompression in a Recompression Chamber for Experiments (110L, Djuro Djakovic, Aparati d.d., Slavonski Brod, Croatia). A-HBO_2_ and 24H-HBO_2_ groups underwent a single session and tissue sampling was performed immediately after decompression or 24 h after the single exposure, respectively. Animals from the 4D-HBO_2_ group underwent one daily session for four consecutive days (intermittent) and tissue sampling was performed on the 5th day 24 h after the last exposure.

### 4.2. Measurement of Blood Pressure

Blood pressure measurement was performed using established protocols, as previously described [18,29,54,55]. Briefly, pressure values were measured using the Spacelabs Medical system (Spacelabs Medical, Inc., Redmond, WA, USA) using the PE-50 catheter inserted into the left femoral artery. The mean arterial pressure was calculated from the obtained values as a sum of systolic and double diastolic pressure and divided by three. As control values, the values measured in A-HBO_2_ animals taken immediately prior to exposure to hyperbaric oxygenation were used. The same animals were used to collect thoracic aorta tissue for further analysis, including real-time quantitative PCR (RT qPCR) and/or protein expression by the Western blot method. Immediately after isolation, the aorta was transferred to a Petri dish with cold saline, cleansed from connective and fatty tissue and frozen in liquid nitrogen and then stored at −80 °C until analysis.

### 4.3. Experiments on Isolated Aortic Rings

The isolated aortic ring experiments were performed according to the well-established protocol in our laboratory [7,8,9,18]. After anaesthesia with ketamine 75 mg kg^−1^ and midazolam 0.5 mg kg^−1^, thoracotomy was made and the thoracic aorta was isolated, cut to a 3–4 mm ring width and then placed in an organic pool (10 mL volume) with Krebs–Henseleit’s solution (solution composition in mmol L^−1^: 120 NaCl, 4.8 KCl, 1.2 KH_2_PO_4_, 2.5 CaCl_2_, 1.2 MgSO_4_, 25.5 NaHCO_3_, 10 glucose and 0.02 EDTA) continuously heated and oxygenated (t = 37 °C, pH = 7.4). After rinsing and stabilization for an hour, the endothelial preservation and maximum contraction tests were done, followed by protocols of acetylcholine-mediated relaxation or hypoxia-mediated protocols, with or without inhibitors.

#### 4.3.1. Acetylcholine-Mediated Relaxation (AChIR)

Following endothelial preservation of the aortic rings and a maximal precontraction test, there was a period of stabilization with noradrenaline (NA 10^−7^ mol L^−1^) precontraction and ACh relaxation at concentrations of 10^−9^ mol L^−1^ to 10^−5^ mol L^−1^, to check NO production and a baseline response to acetylcholine. Acetylcholine-induced relaxation of the aortic rings was measured in the absence and presence of (a) an NO synthase (NOS) inhibitor, Nω-nitro-L-arginine methyl ester (L-NAME, 300 μmol L^−1^), (b) a cyclooxygenase inhibitor, indomethacin (INDO, 10 μmol L^−1^) and (c) a selective CYP450 epoxidase inhibitor, MS-PPOH (10 μmol L^−1^).

#### 4.3.2. Hypoxia-Induced Relaxation (HIR)

After checking the endothelial function and stabilization, the aortic rings were precontracted with norepinephrine (10^−7^ mol L^−1^). Then the Krebs solution oxygenation was replaced by 95% O_2_ + 5% CO_2_ at 0% O_2_ and 5% CO_2_ for 20 min and then switched back to 95 % O_2_ + 5% CO_2_ over 5 min for re-oxygenation. Thereafter, it was washed three times every 2 min followed by 10 min of stabilization and was then incubated with the target inhibitor for 20 min, followed by repeating the hypoxia protocol. The same inhibitors, as well as the acetylcholine-induced relaxation tests, were used in hypoxia-induced vasodilation reactions of aortic rings (L-NAME, (300 μmol L^−1^), INDO (10 μmol L^−1^) and MS-PPOH (10 μmol L^−1^)). Additionally, LW6 transcriptional HIF-1α factor inhibitor was used (HIF-1α inhibitor, 10 mg, Calbiochem, 10^−4^ mol L^−1^).

### 4.4. Relative Gene Expression Determined by RT-qPCR Method

The relative expression of genes relevant to the studied mechanisms was determined by real-time quantitative PCR (RT-qtPCR; real time PCR, Bio Rad CFX96). Homogenization of the sample and total RNA was extracted with TRI reagent (Life Technologies, USA) according to the protocols of Chomczynski et al. [56]. RNA concentration and sample purity were determined using a Nanophotometer P300 UV/VIS, IMPLEN, and confirmation of the RNA presence was performed by placing samples on 1% agarose gel. Sample purification and cDNA preparation were performed according to the manufacturer’s instructions from Sigma-Aldrich and Applied Biosystems.

Expression of following genes was determined: HIF-1α (primer sequence: forward 5’-GCCCAGTGAGAAAGGGGAAA-3’ and reverse 5’-CGGCTGGTTACTGCTGGTAT-3’), cyclooxygenase 1 (COX-1, primer sequence: forward 5’-TCCTGTTCCGAGCCCAGTT-3’ and reverse 5’-GCCAGTGATAGAGGTGGTTGAAT-3’); cyclooxygenase 2 (COX-2, primer sequence: forward 5’-GAAAGAAATGGCTGCAGAGTTGA-3’ and reverse 5’-GCAGGGCGGGATACAGTTC-3’), vascular endothelial growth factor (VEGF, primer sequence: forward 5’-CGACAGAAGGGGAGCAGAAA-3’ and reverse 5’-GCTGGCTTTGGTGAGGTTTG-3’), inducible nitric oxide synthase (iNOS; NOS2, primer sequence: forward 5’-TGGTGAGGGGACTGGACTTT-3’ and reverse 5’-CCAACTCTGCTGTTCTCCGT-3’), endothelial nitric oxide synthase (eNOS; NOS3, primer sequence: forward 5’-CGAACAGCAGGAGCTAGAGG-3’ and reverse 5’-GAGGTGGATCTCTCCTGGGT-3’) and CYP2c11 (primer sequence: forward 5’-CAATCCGCAGTCTGAGTT-3’, reverse 5’-TGCTGAGAATGGCATAAA-3’). Relative expression (Table 2) of a particular gene was quantified in relation to the expression of the hypoxanthine-guanine phosphoribosyl transferase (HPRT, primer sequence: forward 5’-GAAAGAACGTCTTGATTGTTGAAGATAT-3’ and reverse 5’-GAGAGGTCCTTTTCACCAGCAA-3’). 2^−ΔΔCt^ calculation was performed using the Livak and Schmittgen method [57] and presented as fold change for mRNA expressions normalized to housekeeping gene and control (Figure 4).

### 4.5. Relative Protein Expression Determined by Western Blot

The Western blot was made according to the established method of our laboratory [9,55]. The same Western blot protocol was used to determine the COX-1, COX-2, iNOS, HIF-1α, VEGF and CYP2c11 protein expression in the rat aortas, deeply frozen immediately after isolation. For homogenization of the tissue the homogenization buffer (1 mmol L^−1^ EDTA, 10 mmol L^−1^ Tris (Fisher Scientific, Belgium), 0.4% SDS (Acros Organics, USA), cocktail inhibitor protease 0.4 μL/100 μL (Sigma Aldrich, USA)) was used, with addition of 1 mmol L^−1^ cobalt chloride. The frozen tissue was pulverized in liquid nitrogen, weighed and 1 mL of the homogenization buffer was added to 100 mg of tissue. Everything was well-vortexed and centrifuged (15,000× *g*, 30 min, 4 °C) and supernatant was used for further analysis. Before loading on gels, samples were cooked for 5 min in 1:1 ratio with Laemmli sample buffer (2× Laemmli Sample Buffer, Bio-Rad, USA, #161-0737), with the addition of β-mercaptoethanol as a reducing agent. After SDS-PAGE electrophoresis (100V const., approximately 2 h), the samples were transferred to the PVDF (200 mA const., 2 h) membrane, blocked (5% non-fat milk, 1 h, room temperature) and then incubated with the primary antibody (COX-1 rabbit PolyAB, Proteintech Europe, UK, #13393-1-AP, 1:500; COX-2 rabbit PolyAB, Proteintech Europe, UK, #12375-1-AP, 1:500; iNOS rabbit PolyAB, Proteintech Europe, UK, #18985-1-AP, 1:1000; HIF-1α rabbit PolyAB, Thermo Scientific, USA, PAI-16601, 1:1000; VEGF rabbit PolyAB, Proteintech Europe, UK, #19003-1-AP, 1:500; CYP2C11 mouse monoclonal AB, Detroit R&D, USA, #P2C11PT, 1:2000) overnight at 4 °C. The next day, the membrane was incubated with appropriate horse radish peroxidase (HRP)-labeled secondary antibody (goat anti-rabbit HRP, Abcam, UK, ab205718; goat anti-mouse HRP, Santa Cruz Biotechnology, USA, sc-2005, both in 1:7500 dilution) for 2 h at room temperature and was subsequently photographed by a chemiluminescence method using the Pierce ECL Western Blotting Substrate (Thermo Scientific, USA) according to the manufacturer’s instructions, on the Bio-Rad ChemiDoc Imager. As a control of the sample application and for normalization, expression of β-actin (mouse monoclonal antibody, Santa Cruz Biotechnology, USA sc-47778, 1:7500) was determined. 

### 4.6. Determination of CYP450 Enzymatic Pathway Metabolites of Arachidonic Acid 

To determine CYP450 enzymatic pathway metabolites of arachidonic acid in aorta samples, liquid chromatography-tandem mass spectrometry was used [58,59,60,61].

#### Sample Preparation

Blood vessels were placed in to Precellys vials (2 mL), and 1 mL methanol with internal standard (IS) was added (concentration of IS was 50 ng mL^−1^). The samples were homogenized for 2 × 15 s at 4500 rpm and the next 20 s at 6000 rpm in a Precellys Evolution tissue homogenizer (Bertin Technologies, Montigny-le-Bretonneux, France). After homogenization, the samples were centrifuged for 5 min at 10,000 rpm in Thermo SL 40R centrifuge (Thermo Fisher Scientific, Waltham, MA, USA). The supernatants were moved to 1.4 mL polypropylene tubes and 200 μL of DMSO were added. The solvent was completely removed by means of a Biotage SPE DRY 96 sample evaporation system (Biotage AB, Uppsala, Sweden) [62].

Prior to analyses, the samples were reconstituted in a mixture of acetonitrile and water (*v/v*, 1:1), centrifuged and separated in analytical plates (LCMS PLATES: Agilent 96-well plates 0.5 mL polypropylene P/N 5042-1385, J.G. Finneran PTFE 96-Well Pattern Sealing Film, Patented P/N BST-9790).

Standards for calibration curve preparation: arachidonic acid (Sigma-Aldrich, from non-animal source, ≥98.5% (GC)) was dissolved in DMSO (10.0 mg mL^−1^) and diluted in methanol to 100 μg mL^−1^. The Arachidonic Acid CYP450 Metabolite LC-MS Mixture (Cayman Chemicals Company, Ann Arbor, MI, USA) Arachidonic Acid CYP450 Metabolite LC-MS Mixture, Item No. 20665, declared to contain 10 μg mL^−1^ of each compound) was diluted in methanol to 100 μg mL^−1^ (Table 5). The calibration range was from 0.001 to 50 ng mL^−1^ for all of standards except for the arachidonic acid, for which it was from 0.001 to 50 μg mL^−1^. The calibration points were 0.001, 0.005, 0.01, 0.05, 0.1, 0.5, 1, 5, 10 and 50 ng mL^−1^ (μg mL^−1^ for arachidonic acid, respectively). Quantification was performed by summing the three MRM signals of the same transition (differing for 0.001 Da) for each analyte in order to increase sensitivity.

HPLC-electrospray ionization (ESI)/MS-MS: chromatographic separation was performed with a Waters Acquity UPLC^®^ BEH C18 column (2.1 × 50 mm; 1.7 µm particle size) (Waters, USA) and a gradient elution of mobile phase A (0.1% formic acid in water) and phase B (acetonitrile, at a flow rate of 400 µL min^−1^), according to the previously described method, modified as follows [63]. The acetonitrile part was 25% (*v/v*) at first, increasing to 50% within 7.5 min. The percentage of acetonitrile was continuously increased. At 12.5 min it was 60 percent and at 16 min it was 90 percent. It was held at 90% for a further 2 min. At 18.01 min the percentage of acetonitrile was reduced to 25 percent and was maintained at that level for 20 min. The column was reconditioned for 15 min with 75% of 0.1% formic acid in water and 25% acetonitrile (Table 6). Column temperature was 50 °C and autosampler temperature was 4 °C.

**Figure 6:**
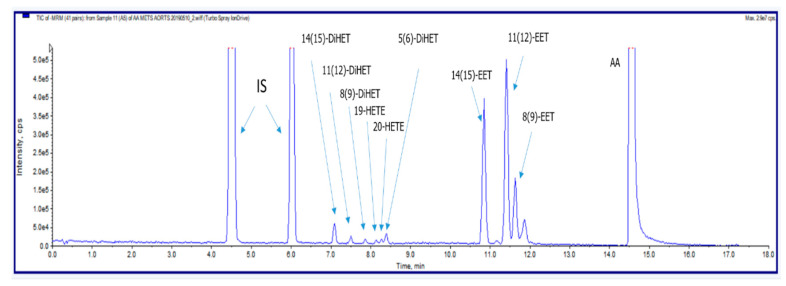
Representative liquid chromatography-tandem mass spectrometry chromatograms illustrating the profile of epoxyeicosatrienoic acid (EET) and HETEs formed by rat cerebral arteries, obtained under described experimental conditions.

All measurements were carried out with a Shimadzu Nexera X2 HPLC system coupled to an AB Sciex QTRAP 6500 ion trap mass spectrometer from AB SCIEX (Framingham, MA, USA), operated under negative-ion electrospray (ESI) conditions in the SIM (Single (Selected) Ion Monitoring) and MRM (Multiple Reaction Monitoring) modes, respectively. Representative liquid chromatography-tandem mass spectrometry chromatograms are presented in Figure 6.

### 4.7. Statistical Analysis

All results are expressed as average ± standard deviation (SD). *p* < 0.05 was considered statistically significant. Acetylcholine-induced relaxation is expressed as a percentage of the maximum contraction. The response to ACh was analyzed via a two-way ANOVA with a post hoc Bonferoni test. For a comparison of mRNA and protein expression results and hypoxia-mediated relaxation of aortic rings, a one-way ANOVA variance test was used, or in the case of unequal distribution, Holm–Sidak or Kruskal–Wallis test data were obtained. For individual results, the determination of the difference between the normal distribution of numeric variables between the two independent groups was performed using Student’s *t*-test, and in the case of a deviation from the normal distribution, the Mann–Whitney U test was used. SigmaPlot v.12 (Systat Software, Inc., Chicago, IL, USA) and GraphPadPrism, Version 5.00 for Windows, GrafPad Software (San Diego, CA, USA) were used for statistical analysis.

## 5. Conclusions

In conclusion, intermittent HBO_2_ exposure causes enhanced relaxation, most likely by activating the CYP450 epoxygenases pathway of arachidonic acid metabolism and by increasing the formation of and sensitivity to EETs. In addition, HBO_2_ increases the expression of HIF-1α, which stimulates COX pathway expression and prostacyclin formation, thereby mediating enhanced relaxation in intermittent HBO_2_ exposure.

## Figures and Tables

**Table 1:** Measurements of body mass and mean arterial pressure.

	CTRL	A-HBO_2_	24H-HBO_2_	4D-HBO_2_
Body mass [g]	329.4 ± 18.25	332.4 ± 21.53	318.6 ± 29.62	323.20 ± 39.49
Mean arterial pressure [mmHg]	107 ± 4	82 ± 5 *	107 ± 9	105 ± 3

* Results are shown as mean ± SD (standard deviation); *n* = 6 (number of rats per group), * *p* < 0.05 compared to CTRL group. CTRL–untreated group; A-HBO_2_–group receiving a single HBO_2_ exposure; 24H-HBO_2_–group examined 24 h after single exposure; 4D-HBO_2_–group receiving four consecutive days of single HBO_2_ exposure.

**Table 2:** Relative mRNA expression of cyclooxygenase 1 and 2 (COX 1 and COX 2), nitric oxide synthase (iNOS and eNOS), hypoxia inducible factor 1 alpha (HIF-1α), vascular endothelial growth factor (VEGF) and CYP2C11 in rat aorta normalized to *HPRT* housekeeping gene.

	CTRL	A-HBO_2_	24H-HBO_2_	4D-HBO_2_
COX-1	4.20 ± 1.49	4.42 ± 1.87	1.74 ± 0.59 *^†‡^	3.53 ± 0.88
COX-2	1.18 ± 0.78	1.45 ± 0.88	0.47 ± 0.33 ^†^	0.33 ± 0.37 *^†^
eNOS	0.13 ± 0.12	0.03 ± 0.03	0.13 ± 0.13	0.04 ± 0.03
iNOS	0.38 ± 0.21	0.11 ± 0.04 *	0.12 ± 0.08 *	0.21 ± 0.11
HIF-1α	0.38 ± 0.31	0.48 ± 0.33	0.82 ± 0.28 *^†^	0.75 ± 0.18 *^†^
VEGF	0.19 ± 0.40	0.30 ± 0.26	0.53 ± 0.24 *^†^	0.58 ± 0.62 *^†^
CYP2c11	1.11 ± 0.45	0.69 ± 0.26	0.55 ± 0.29	2.21 ± 1.09 *^†‡^

Data are presented as mean ± SD (standard deviation); *n* = 6 (number of rats per group). * *p* < 0.05 compared to CTRL group; † *p* < 0.05 compared to A-HBO_2_ group; ‡ *p* < 0.05 compared to 24H-HBO_2_ group.

**Table 3:** Liquid chromatography-tandem mass spectrometry (LC/MS–MS) determination of CYP450 enzymatic pathway vasodilating 11(12)-epoxyeicosatrienoic acid (EET), 14(15)-EET, 8(9)-EET and corresponding DiHETs and vasoconstriction-causing metabolite (20- hydroxy-eicosatrienoic acid (HETE)) of arachidonic acid in aortic tissue.

	CTRL	A-HBO_2_	24H-HBO_2_	4D-HBO_2_
AA-259 [μg g^−1^]	60.4 ± 20.39	48.7 ± 23.72	89.3 ± 34.19 ^†^	45.4 ± 17.23
11 (12)-EET [ng g^−1^]	0.57 ± 0.17	0.63 ± 0.26	0.46 ± 0.25	0.39 ± 0.16
14 (15)-EET [ng g^−1^]	0.49 ± 0.11	0.49 ± 0.23	0.47 ± 0.26	0.35 ± 0.22
20-HETE [ng g^−1^]	3.94 ± 1.59	6.43 ± 1.65 *^†^	4.64 ± 2.68	2.94 ± 1.51
14 (15)-DiHET [ng g^−1^]	6.87 ± 0.92	9.84 ± 2.45 *	9.83 ± 2.11 *	7.51 ± 1.10
11 (12)-DiHET [ng g^−1^]	2.62 ± 0.29	4.92 ± 1.74 *	4.13 ± 1.30 *	3.08 ± 0.53
5 (6)-DiHET [ng g^−1^]	1.23 ± 0.41	1.12 ± 0.51	1.39 ± 0.23 ^†^	1.08 ± 0.13
8(9)-EET [ng g^−1^]	0.98 ± 0.17	1.41 ± 0.35 *^†^	1.50 ± 0.20 *^†^	0.88 ± 0.36
8 (9)-DiHET [ng g^−1^]	1.67 ± 0.32	5.14 ± 0.94 *	4.75 ± 1.62 *	3.45 ± 1.77

* Data are presented as mean ± SD (standard deviation); *n* = 5; * *p* < 0.05 compared to CTRL group; † *p* < 0.05 compared to 4D-HBO_2_ group.

**Table 4:** Ratios of vasodilatory to vasoconstrictory CYP450 AA metabolites in aortic tissue.

	11 (12)-EET/20 HETE	14 (15)-EET/20 HETE	8 (9)-EET/20 HETE	11 (12)-DiHET/20HETE
CTRL	0.158 ± 0.107	0.149 ± 0.078	0.317 ± 0.099	0.885 ± 0.391
A-HBO_2_	0.098 ± 0.035	0.076 ± 0.033 *^†‡^	0.173 ± 0.055 *^†‡^	0.760 ± 0.179 *^†‡^
24H-HBO_2_	0.146 ± 0.084	0.131 ± 0.076	0.323 ± 0.173	1.228 ± 0.595
4D-HBO_2_	0.166 ± 0.107	0.151 ± 0.095	0.312 ± 0.187	1.313 ± 0.758 *

* Data are presented as mean ± SD (standard deviation); *n* = 5. * *p* < 0.05 compared to CTRL group; † *p* < 0.05 compared to 24h-HBO_2_ group; ‡ *p* < 0.05 compared to 4D-HBO_2_ group.

**Table 5:** List of standards used for liquid chromatography-tandem mass spectrometry.

Analyte	Units	RT (min)	MRM Transition
14(15)-DiHET	ng mL^−1^	7.09	337.100/207.100 Da
11(12)-DiHET	ng mL^−1^	7.50	337.100/167.100 Da
8(9)-DiHET	ng mL^−1^	7.87	337.100/185.100 Da
19-HETE	ng mL^−1^	8.10	319.095/231.300 Da
20-HETE	ng mL^−1^	8.27	319.095/245.300 Da
5(6)-DiHET	ng mL^−1^	8.39	337.100/145.100 Da
14(15)-EET	ng mL^−1^	10.8	319.143/219.100 Da
11(12)-EET	ng mL^−1^	11.4	319.113/167.100 Da
8(9)-EET	ng mL^−1^	11.6	319.113/127.100 Da
AA	μg mL^−^^1^	14.5	303.300/259.300 Da

**Table 6:** Gradient table for chromatographic separation (Mobile phase A: 0.1% formic acid in water; mobile phase B: acetonitrile, %B–percentage of acetonitrile as mobile phase).

Time [min]	%B
0.10	25
7.5	50
12.5	60
16	95
18	95
18.01	25
20	25

## Abbreviations

CYP450	Cytochrome P450
HIF-1α	Hypoxia inducible factor 1 alpha
AChIR	Acetylcholine-induced relaxation
HIR	Hypoxia-induced relaxation
HBO_2_	Hyperbaric oxygen
EET	Epoxyeicosatrienoic acid
HETE	Hydroxyeicosatetraenoic acid
COX-1	Cyclooxygenase 1
COX-2	Cyclooxygenase 2
AA	Arachidonic acid
LC-MS/MS	Liquid chromatography-tandem mass spectrometry
NO	Nitric oxide
ANGII/ANG(1-7)	Angiotensin II/Angiotensin (1-7)
PGI2	Prostacyclin
cAMP	Cyclic adenosine monophosphate
ROS	Reactive oxygen species
RNS	Reactive nitrogen species
MS-PPOH	N-(methyl sulfonyl)-2-(2-propynyloxy)-benzenehexanamide
INDO	Indomethacin
L-NAME	Nω-nitro-L-arginine methyl ester
iNOS	Inducible nitric oxide synthase
eNOS	Endothelial nitric oxide synthase
VEGF	Vascular endothelial growth factor
mRNA	Messenger ribonucleic acid
EDHFs	Endothelium-derived hyperpolarizing factors
ACh	Acetylcholine
SD	Sprague-Dawley rats
RT qPCR	Quantitative reverse transcription polymerase chain reaction
EDTA	Ethylenediaminetetraacetic acid
RNA	Ribonucleic acid
HPRT	Hypoxanthine-guanine phosphoribosyl transferase
SIM	Single (Selected) Ion Monitoring
MRM	Multiple Reaction Monitoring
SDS	Sodium dodecyl sulfate
SDS-PAGE	Sodium dodecyl sulfate polyacrylamide gel electrophoresis
DMSO	Dimethyl sulfoxide
HPLC-ESI/MS-MS	High-performance liquid chromatography/electrospray ionization tandem mass spectrometry
